# A Personalized eHealth Transition Concept for Adolescents With Inflammatory Bowel Disease: Design of Intervention

**DOI:** 10.2196/12258

**Published:** 2019-04-24

**Authors:** Katrine Carlsen, Mette Hald, Marla C Dubinsky, Laurie Keefer, Vibeke Wewer

**Affiliations:** 1 Department of Pediatrics Hvidovre University Hospital Hvidovre Denmark; 2 Division of Pediatric Gastroenterology and Hepatology Susan and Leonard Feinstein IBD Center Icahn School of Medicine at Mount Sinai Hospital New York, NY United States; 3 Division of Gastroenterology Susan and Leonard Feinstein IBD Center Icahn School of Medicine at Mount Sinai Hospital New York, NY United States

**Keywords:** inflammatory bowel disease, adolescents, transition, transfer, adult care

## Abstract

**Background:**

Transfer from pediatric to adult care is a crucial period for adolescents with inflammatory bowel disease (IBD).

**Objective:**

Our aim was to develop a personalized transition-transfer concept including relevant tools in an established eHealth (electronic health) program.

**Methods:**

Required transition skills and validated patient-reported outcome measures (PROMs) were identified via bibliographic search and clinical experience and were implemented into an existing eHealth program.

**Results:**

The following skills were identified: disease knowledge, social life, disease management, and making well-informed, health-related decisions. The PROMs included the following: self-efficacy (the IBD Self-Efficacy Scale—Adolescents), resilience (the 10-item Connor-Davidson Resilience Scale), response to stress (the Child Self-Report Responses to Stress—IBD), and self-management and health care transition skills (the Self-Management and Transition to Adulthood with Treatment questionnaire). Starting at age 14, the patient will be offered a 1-hour annual transition consultation with an IBD-specialized nurse. The consultation will be based on the results of the PROMs and will focus on the patient's difficulties. Patients will complete the PROMs on the eHealth program at home, allowing nurses and patients to prepare for the meeting. Symptom scores and medication will be filled out on the eHealth program to support disease self-management. The consultation will be a topic-centered dialogue with practical exercises. During routine outpatient visits with the provider, parents will be left out of half of the consultation when the patient is 16 years old; at 17 years old, the parents will not be present. At the transfer consultation, the pediatric provider, the adult gastroenterologist, the pediatric nurse, the patient, and the parents will be present to ensure a proper transfer.

**Conclusions:**

We have conducted a personalized eHealth transition concept consisting of basic elements that measure, train, and monitor the patients' transition readiness. The concept can be implemented and adjusted to local conditions.

## Introduction

The incidence of inflammatory bowel disease (IBD)—primarily represented by the diagnoses ulcerative colitis and Crohn´s disease—is increasing among children and adolescents [1-6]. Consequently, in the coming years there will be a larger burden on adult IBD care settings to manage the complex needs of patients transferred from pediatric IBD departments [7-9]. Ensuring that the patients and parents are prepared for the transfer, specifically the *physical handover* of patients from one setting to another, as well as receiving the patients after the transfer, is a challenge for both the pediatric and adult gastroenterological staff [10]. One of the most costly periods for IBD care is in the first year after transfer, from pediatric to adult care; the costs during this period are largely preventable, driven by unplanned hospitalizations, relapse, and nonattendance at scheduled appointments [7-9].

Different approaches have been used over time to improve the transfer process. These approaches have included structured discharge summaries provided on behalf of the pediatric department for the convenience of the adult department and/or joint consultations. Consultations include the patient, the parents, the nurse, and the adult and pediatric provider focused on facilitating the referral between providers in a *warm* handoff [11]. This approach is insufficient. Studies in adolescents and young adults with other chronic illnesses suggest that in order to improve disease outcomes and reduce health care costs, care must include (1) coordination provided over time, (2) communication between the patient and at least one member of the care team, and (3) skills-based training [12]. Transition care is an extended period of patient empowerment that equips adolescents (10-19 years old) [13] with the skills and knowledge necessary to manage their own health and well-being as they move from pediatric to adult-centered services. This is a more comprehensive approach to addressing the needs of adolescents and young adults with IBD. The process requires a coordinated, team-based shifting of responsibilities of disease self-management from the parent to the patient [14]. Less is known about how to best leverage technology to drive delivery of personalized skills training to a transitioning patient prior to entering adult-centered care [15].

**Figure 1:**
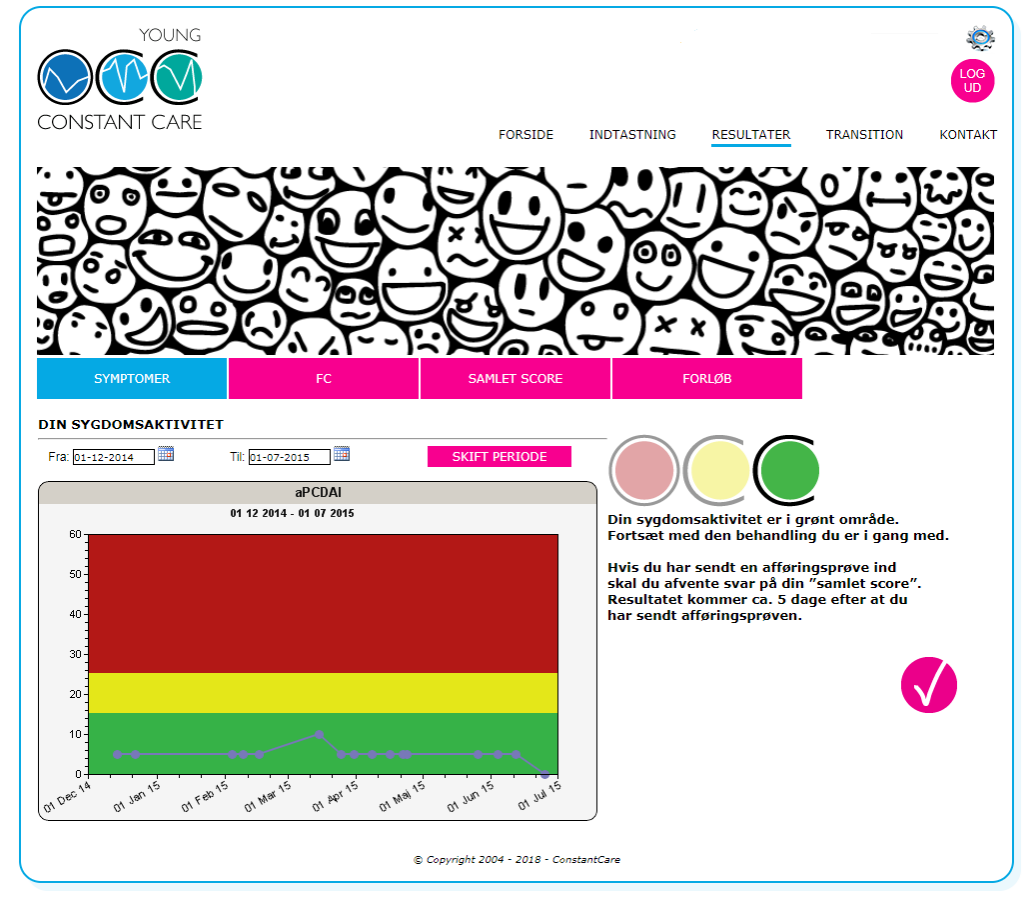
Screenshots from the website Young Constant Care (Danish) [16]. The disease activity is tracked over time; the traffic light color code is used to visualize disease activity based on symptom index. Patients are provided with a guideline regarding the disease activity (right-hand side of screenshot).

We have previously demonstrated and published that the use of the interactive eHealth (electronic health) solution Young Constant Care [16], designed for adolescents (10-17 years old) with IBD, is usable in this age group [17,18]. An essential element of eHealth is the use of patient-reported outcome measures (PROMs) registered in real time due to the Internet connection, which allows timely relevant monitoring and personalized treatment to the individual patient. Specifically, patients register symptoms through the Pediatric Ulcerative Colitis Activity Index or abbreviated Pediatric Crohn´s Disease Activity Index [19,20] and their type of medication. The symptom score is presented for the patient as a curve with traffic light zones (ie, green, yellow, and red colors) to indicate whether the disease activity is mild, moderate, or severe (see Figure 1). The traffic light acts as feedback to the patient on his or her symptoms and a guide to the patient to interpret how severe the disease activity is at a given time. Registration of medication helps the patient to remember and be aware of his or her own treatment progress. On an administration page only seen by the IBD team, all patients are listed and the staff can access the scores from the patients. Participants using our eHealth program were empowered and educated to be responsible for reporting symptoms themselves instead of relying on their parents [17].

The disease activity is tracked over time and the traffic light color code is used to visualize disease activity based on symptom index. On the right-hand side of the screenshot in Figure 1, the patients are provided with a guideline regarding the disease activity.

Based on our previous results involving adolescent patients in self-managing their disease through eHealth, it was natural to extend this program beyond reporting of IBD symptoms to include assessment and remediation of other critical self-management skills. We wished to personalize patients’ transition preparedness by involving patients actively in their own transition process. Specifically, our aim was to develop a transition-transfer concept that included incorporation of validated measures of transition preparedness in the established eHealth program.

## Methods

### Development of the Transition Concept

The structure of the transition concept was developed in cooperation and agreed upon by two pediatric IBD centers to improve the ability of adapting the concept, regardless of cultural diversions. One center was located in Europe—Department of Pediatrics, Hvidovre University Hospital, Hvidovre, Denmark—and one was located in North America—Division of Pediatric Gastroenterology and Hepatology, Susan and Leonard Feinstein IBD Center, New York, United States.

### Identification of Transition Skills

Identification of needed transition and transfer skills and expectations was based on a bibliographic search and clinical experience. The bibliographic search was performed without a date restriction in June 2016 on the online database PubMed. Medical Library Subject Heading (MeSH) terms and keywords were created by combinations of “inflammatory bowel disease” with the subjects “transition to adult care,” “education,” and “skills” using “AND.” Relevance was determined by screening titles, abstracts, or full-text publications. Reference lists of relevant articles were screened for further potentially relevant studies. A total of 125 publications were returned using the initial search and 28 were excluded based on their abstracts or full text. After retrieving duplications, 40 original publications remained. The types of publications were as follows: randomized clinical trial (n=1); intervention trials (n=3); investigations of the patients’ transition readiness (n=13); investigations of providers’ transition experience and opinions (n=6); reviews of the literature (n=8); and transition recommendations (ie, needs and concerns) (n=9). Domains were extracted and transformed to a *transition readiness checklist* for the pediatric provider and a *transfer readiness checklist* for the adult provider to assess the patient’s transition and transfer progress, respectively (see Multimedia Appendices 1 and 2).

### Patient-Reported Outcome Measures

The US Food and Drug Administration defines patient-reported outcomes as any report of the status of a patient's health condition that comes directly from the patient, without interpretation by a provider or anyone else [21]. In this context, PROMs [22] are questionnaires that evaluate the patient´s self-reported capability and performance of transition readiness; these were identified in this study. The requirements of the PROMs were that they needed to be previously validated and usable in international settings; the questionnaires also had to be of an accepted length (ie, maximum 20 items). Furthermore, the total number of questionnaires were limited in order to ensure the quality of the answers. The agreement of included PROMs was performed by a consensus group (ie, consultants, physicians, psychologists, and nurses). PROMs existing in nonnative languages were translated by the cross-cultural adaptation translation method (ie, forward translation, backward translation, and cognitive testing) after agreement and approval with the developers of the PROMs [23]. Selected PROMs were implemented within the existing eHealth program Young Constant Care [16].

### Approval

Young Constant Care [16] meets the requirements of the EU General Data Protection Regulation (May 2018) and is hosted on a server owned by the Capital Region, Denmark. Approval from the Danish Ethical Committee and Data Protection Agency will be obtained before enrollment of patients.

## Results

### Four Components of the Transition Concept

#### Component 1: Preparation of the Annual Consultation (eHealth and Face-to-Face)

The transition concept offers the patient an annual 1-hour transition consultation with a pediatric IBD-specialized nurse. The purpose of the transition consultation is to improve transition skills and to discuss individual difficulties. Patients will complete the questionnaire on the eHealth webpage Young Constant Care [16], to which the patients are provided access in advance through a personal double log-in—personal password and a code sent to the patient´s phone. The webpage is designed to work on mobile phones, tablets, and laptops. Completing the PROMs prior to the consultation allows patients to be prepared for the topics around which the consultation should be centered. It also allows the nurse to prepare the consultation based on the patient´s answers to the selected PROMs addressing the patient´s abilities and difficulties (ie, the patient’s score). The PROMs are presented below in the *Specific Contents of the Transition Concept: Transition Skills and Patient-Reported Outcome Measures* section. The actual topic of each consultation will be defined in collaboration between the patient and the nurse at the beginning of each consultation, leading to an individualized topic-centered dialogue with practical exercises (see Table 1).

The eHealth transition concept also provides the patient with mind-body techniques of progressive muscle relaxation, relaxation imagery, and deep breathing exercises to cope with pain and fatigue, which are often present despite relevant treatment [24]. If the patient presents substantial psychological difficulties, the patient can be referred to a psychologist. Nurses are supervised in regular 6-month intervals by a health psychologist, allowing the nurses to evaluate and reflect on the themes of the conversations in the transition consultation and to discuss the challenges encountered during the sessions. The disease knowledge is not systematically evaluated by tests, but is discussed during the regular conversation and consultation with the nurse and the provider.

#### Component 2: Regular Consultations With the Provider (Face-to-Face)

Aiming to prepare and train the patient in disease management, the patient will register his or her symptoms at home prior to the consultation with the provider, interpret his or her disease activity, and record current medications in the eHealth program Young Constant Care [16]. Starting at age 14, the consultation with the provider in the outpatient clinic will change from being oriented toward the parents to being primarily oriented toward the patient. Once the patient is 16 years old, parents will only take part in half of the consultation; when the patient is 17 years old, the parents will no longer participate. This may be a challenge for both the patient, parent, and pediatrician and the rationale for this step should be addressed at the previous consultation.

#### Component 3: Transfer Consultation (Face-to-Face)

At a joint consultation when the patient is 18 years old, the pediatric provider, the adult gastroenterologist, the pediatric nurse, the patient, and the patient’s parents will participate. At the joint consultation, the adult gastroenterologist will explain the structure and the approach of the adult department and, going forward, how the consultations will be conducted. Afterward, the adult gastroenterologist will receive a summary of the disease history in the medical journal and the patient will receive a status letter.

#### Component 4: Evaluation

**Figure 2:**
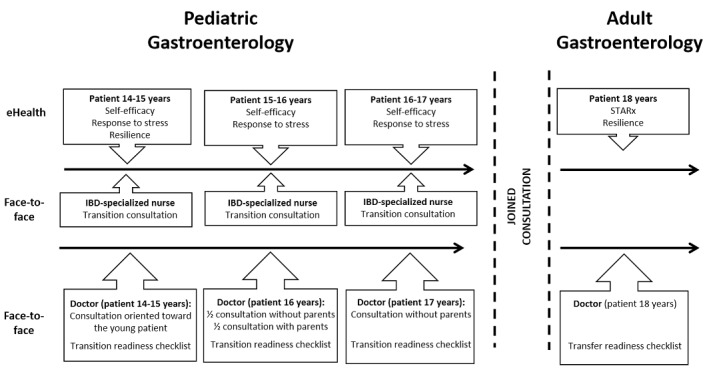
Flowchart of the transition-transfer concept. IBD: inflammatory bowel disease; STARx: Self-Management and Transition to Adulthood with Treatment.

Progression in PROMs will be evaluated by comparing the repetitive scores from the patients. The patient´s answers to the questionnaires will be stored in the eHealth program. The patient will complete the Self-Management and Transition to Adulthood with Treatment (STARx) questionnaire at the time of transfer. The pediatric provider will assess the patient annually using the *transition readiness checklist* (see Multimedia Appendix 1) and the gastroenterologist will assess the patient at transfer using the *transfer readiness checklist* (see Multimedia Appendix 2). The triple assessment results in a comprehensive evaluation, which is needed, as transition readiness is multidimensional and is biased by the assessor. The transition consultations with IBD nurses will be documented systematically in the patient´s medical record. The flowchart of the concept is presented in Figure 2.

**Table 1:** Exercises to improve the patient´s transition skills.

Topics	Exercises (requests and items to address by the IBD^a^-specialized nurse)^b^
IBD knowledge	Explain the disease for me. Mark on a drawing of the intestinal system where your disease is located. Review your onset of diagnosis, treatment history, and surgeries. Explain what symptoms you should be aware of. Explain what to do during a relapse. Where can you gather additional information about the disease? Information from the nurse: significance of alcohol and drugs on the disease. Information from the nurse: IBD in relation to sex, contraception, fertility, and pregnancy.
Management of contact with the hospital	Explain how to contact the hospital and how to make a new appointment in the outpatient clinic. Prepare a list of topics you can reference prior to your outpatient visits. Make a list of phone numbers and contact information for the hospital.
Medication	Explain what medication your treatment consists of (ie, type, dose, and when to take the medication). Explain how to renew prescriptions. Are you aware of how to travel with your medication? Are you aware of what travel documentation you need?
Medical adherence	Identification of the obstacles to good adherence (ie, forgetful, self-conscious, uncomfortable, and belief by patient that “it doesn´t work anyway”). Solution to improve adherence (ie, use of alarm, pill box, and goals).
Worries	Conversation concerning what is difficult in relation to the disease. Define beforehand who you would like to inform about the disease. Prepare sentences about the disease to be used in new relationships and among acquaintances. Conversation regarding feeling different from peers. Conversation regarding how the family manages the disease. Challenges related to absence from school and work.
Network: family and friends	Are you aware of whom you can rely on if or when the disease progresses? Write the names of the persons that you can place in your “inner circle” and “peripheral circle” of relationships.
Stress, pain, and fatigue	Relaxation exercise and visualization Breathing exercise Mindfulness

^a^IBD: inflammatory bowel disease.

^b^The exercises are performed in cooperation with the IBD-specialized nurse. Selected topics depend on the patient´s difficulties, which are partially determined from the patient’s responses to the patient-reported outcome measures (PROMs).

### Specific Contents of the Transition Concept: Transition Skills and Patient-Reported Outcome Measures

#### Overview

Based on the bibliographic search and discussion among the consensus group, the following skills were found to be important for adolescents to achieve in the transition phase [25]:

1. Disease knowledge (eg, regarding the diagnosis, medication, adherence, hospital procedures, and symptoms).
2. Disease self-management (eg, independently report to the provider, recognize and react on a flare, refill of prescriptions, and coping with the disease).
3. Management of social challenges (eg, social challenges related to the disease, who to rely on, and missed school and work because of the disease).
4. Taking part in health-related decisions in cooperation with the provider.

Therefore, the existing eHealth program Young Constant Care [16] was expanded with an implementation of four selected PROMs that evaluated the patient´s self-reported capability and performance of transition readiness: disease knowledge, disease self-management, management of social challenges, and taking part in health-related decisions.

#### Self-Efficacy: The Inflammatory Bowel Disease Self-Efficacy Scale—Adolescents

The construct of self-efficacy describes a person´s perception of his or her own ability to manage skills required to master new challenges and has proven to be related to their level of self-management [26]. The disease-specific, self-reported, IBD Self-Efficacy Scale—Adolescents [26,27] questionnaire has been developed in cooperation with adolescents with IBD and covers skills related to the challenges of living with IBD between the ages of 12 and 25 years old. During prior work investigating transition readiness, we found self-efficacy to be a predictor [28]. The questionnaire is centered on confidence in various self-management tasks: managing medical care, managing everyday life with IBD, managing feelings, and managing the future with IBD. Answers are given on a 5-point Likert scale and a higher score represents increased self-efficacy (range 13-65) [26,27].

#### Resilience: The 10-Item Connor-Davidson Resilience Scale

Resilience refers to a person´s capability to restore good functioning after exposure to stress or trauma; for instance, how well a person “bounces back” after a diagnosis of IBD [29,30]. The results of adaptation to a given exposure can either improve, neutralize, or overload functioning of a person leading to different levels of dysfunction. The 10-item Connor-Davidson Resilience Scale [29] is a validated measure of the resilience construct [29]; in previous work, we have found this measure to be a good predictor of transition readiness [28]. The topics of the questionnaire are centered on how to adapt and handle changes and the capability to achieve goals despite obstacles. Answers are given on a 5-point Likert scale, ranging from 0 (*not true at all*) to 4 (*true nearly all the time*), and a higher score represents increased resilience (range 0-40) [29,30].

#### Response to Stress: The Child Self-Report Responses to Stress—Inflammatory Bowel Disease

The impact of a stressor on an individual is mitigated by his or her coping mechanisms. The Child Self-Report Responses to Stress—IBD questionnaire [31,32] was developed specifically for children and adolescents with IBD and covers topics that the patients may find stressful or difficult to deal with. The items cover coping and stress responses related to control engagement (eg, problem solving and positive thinking), disengagement coping (eg, avoidance), involuntary engagement (eg, physiological arousal), and involuntary disengagement (eg, emotional numbing). This questionnaire consists of 10 items; answers are given on a 4-point Likert scale (ie, *none, a little, some,* or *a lot*) [31]. The score is calculated using a scoring system delivered by the developer.

#### Self-Management and Health Care Transition Skills: The Self-Management and Transition to Adulthood With Treatment Questionnaire

The STARx [33] is a generic self-reported questionnaire that measures self-management and health care transition skills in adolescents with a chronic disease who are in current treatment [34]. The items are divided into the following factors: medication management, provider communication, engagement during appointments, disease knowledge, adult health responsibilities, and resource utilization. Answers are given on a 5-point Likert scale and a higher score represents an increased transition readiness.

## Discussion

### Principal Findings

We have developed a personalized transition concept including relevant and concrete measures involving the patient, provider, and nurse. Transition elements (ie, PROMs and exercises) are implemented in an existing eHealth program. The current transition concept is presented by specific transition skills that are needed; exercises on how to improve the patient´s skills by identification of difficulties via PROMs can be adapted to other sites. The adaptation can be implemented with and without access to an eHealth tool, as the concept can be managed nondigitally (ie, use of paper) as well. The concept has been made ready for testing in clinical practice.

Skills and habits learned during the years of adolescence to manage their own disease and social challenges related to the disease form the basis of the patients’ behavior in adulthood [35]. Transition readiness is one of the factors affected by the patients’ coping strategies and how they adapt to new circumstances and cultural differences. Therefore, there is a need to approach the transition phase in a holistic and individual manner and to provide patients with appropriate coping strategies that can support them later in life.

A few intervention studies have investigated the impact of a different approach to improve transfer readiness, of which joint consultations were an essential part [36-39]. In a study by Cole et al [36], disease outcomes were evaluated retrospectively according to joint consultations (ie, pediatric provider, adult gastroenterologist, and nurse), which were offered to the patient beginning at the age of 15. At the joint consultation, disease-specific education and information were provided to the patients and parents. In a study by Yerushalmy-Feler et al [38], patients were offered three appointments during a 6-month period in a transition clinic offering joint consultation at the age of 17. The three appointments focused on (1) introduction to the transition (2), improvement of IBD knowledge and self-management, and (3) summation and short-term and long-term planning. Both studies reported improvements in outcome—disease activity and self-efficacy, respectively—after participation in the transition clinic. Yerushalmy-Feler et al [38] evaluated patients’ self-efficacy using the IBD-Yourself questionnaire [40] at baseline and at the end of the intervention; however, no monitoring of transition readiness progress was implemented, neither by the providers nor the patient. Cole et al [36] monitored transition readiness using nonvalidated tools by the provider but did not involve PROMs. In our opinion, it is essential to regularly monitor the transition process for both the patient and provider, in order to optimize the process and individualize the effort.

Providing the right support can be a difficult task as it is complex to measure patients’ transition readiness. Different questionnaires, such as the Transition Readiness Assessment Questionnaire [41] and the STARx questionnaire, have been used widely; however, no gold standard exists. Therefore, it seems that an appropriate solution is to involve both the pediatric assessment (ie, *Transition readiness checklist*) and the adult provider’s assessment (ie, *Transfer readiness checklist*), as well as the patient’s view (ie, STARx) on the situation to ensure a comprehensive evaluation of the patient’s transition readiness, thereby gaining a measure of the transition concept´s effectiveness.

### Systematic Approach

The regular consultations with the provider are often busy, with limited time per consultation, which emphasizes the need for an incorporated systematic approach to ensure a proper individual transition and transfer process for all patients. However, the concept needs to embrace individualities and the ideal number of pretransfer consultations should be flexible depending on the patient’s needs. The use of PROMs can help focus resources on the patients who need it most; however, access to a multidisciplinary team of providers and an economic surplus are needed.

### Advantages of Technology

Using an Internet-based tool to collect questionnaires has advantages. This is true for both for the patient, who can prepare for the consultation at home, and for the providers, since results from the PROMs, which are available beforehand, can be implemented in the consultation. Information and exercises can easily be accessed through an Internet platform. The eHealth program is future oriented and intended to be managed by the adolescent who must report symptoms and medication changes in the program as part of building his or her involvement in their own disease management. This facilitates and promotes the competencies and management skills of the patient, enabling him or her to gradually take full responsibility for the management of their disease and allowing the parents to step back. The challenge is to establish an eHealth system and to ensure that the system is in accordance with standards that cybersecurity officials request.

### Perspectives

Our transition concept focuses on the patient, IBD nurse, and provider. However, two components could receive further attention. First, more attention should be paid to the parents and the need for information about how best to promote independence in their adolescent child and how to gradually relinquish responsibility and let go [42]. Second, a posttransfer adaption environment should be provided in the adult gastroenterological department, acknowledging that the patients may not be completely ready for transfer at age 18 [10]. This is especially relevant in countries with public health services, where postponing transfer is not a possibility for economic reasons [15].

### Strengths and Limitations

This transition concept was developed by an interdisciplinary team involving two IBD centers. The strength of the concept is the inclusion of both the provider’s and the patient´s evaluation of the transition process, acknowledging the strength of implementing and using PROMs. All self-reported components in the concept are validated and translated from English to Danish through a systematic translation process involving cognitive tests by the target group (ie, adolescent patients). However, a limitation is that we did not involve patients and parents in the selection of the PROMs. The involvement of the user (ie, the patient) in the design phase has been shown to be important and to improve the usability of the concept. Patients will be invited to evaluate the concept after use and, based on their evaluation, changes can be implemented.

Despite the transition and transfer checklists being based on existing knowledge of selected topics that dominate the literature, they are limited by a lack of validation. The effectiveness of the concept will be evaluated after implementation in the clinic. The search strategy of the literature to identify transition skills was limited by the use of only one database, and that search was not validated by multiple reviewers.

### Conclusions

In conclusion, we have developed a personalized transition concept consisting of basic core elements, which can be implemented and adjusted to local conditions. The concept involves transition consultations with an IBD-specialized nurse and ongoing evaluation from both the patient and provider. The transition concept is designed to run for a long period of time for each patient—3 years—to ensure proper preparedness before transfer.

The transition concept will be evaluated at the Department of Pediatrics, Hvidovre University Hospital, Hvidovre, Denmark, in an interventional case-control study (ie, patients enrolled in the transition concept intervention group versus patients without transition preparedness). We hypothesize that the intervention group will achieve a higher transition and transfer readiness score that the control group at the time of transfer as assessed by PROMs and observer-reported outcomes. We also hypothesize that the intervention group will show improved disease management and disease course the first year following transfer. Participants will include IBD patients, 14-18 years old: an intervention group, 14-16 years old, and a control group, 17-18 years old, naïve to the transition concept. All patients will be followed until the age of 19.

## Appendix

Multimedia Appendix 1 Transition readiness checklist for the pediatric provider.

Multimedia Appendix 2 Transfer readiness checklist for the gastroenterological provider in adult care.

## Abbreviations

eHealth: electronic health
IBD: inflammatory bowel disease
MeSH: Medical Library Subject Heading
PROM: patient-reported outcome measure
STARx: Self-Management and Transition to Adulthood with Treatment
