# Supplementary material for: A Personalized eHealth Transition Concept for Adolescents With Inflammatory Bowel Disease: Design of Intervention
Source: JMIR Pediatr Parent. 2019 Apr 24;2(1):e12258. doi: 10.2196/12258 (PMC6715343; doi:10.2196/12258)
Supplement: Multimedia Appendix 1 [file pediatrics_v2i1e12258_app1.pdf]

Multimedia Appendix **1: Transition readiness checklist for the pediatric provider.**

Each item is assessed according to how independently managed the skill is on a 4-point Likert scale: Cannot manage / perform with lot of guidance / perform with little guidance / perform independently.

ITEMS

**KNOWLEDGE**
The patient -<br>1. can explain own diagnosis and can briefly tell about disease history<br>2. can name his/her current medication (regimen and dose/number of pills)<br>3. can explain the purpose and main side effects of current medication<br>4. can explain the nature of a disease flare<br>5. can explain the impact of non-adherence<br>6. can explain the rationale for checking blood samples, fecal samples and undertaking colonoscopy<br>7. know where to obtain further information about IBD<br>8. know how alcohol and drugs impacts the disease and is aware of contraceptives/safe sex<br>9. has knowledge about the transition process<br>10. has knowledge about the impact of diet on disease and symptoms
**SELF-MANAGEMENT**
The patient –<br>11. can report symptoms to a health care professional<br>12. can manage treatment<br>13. can recognize and handle a flare and knows how to get help<br>14. can communicate with the provider; has prepared questions for the consultation<br>15. can make a new appointment; know how to get in contact with the health-care team if needed<br>16. remember to refill medication and ask for prescription when needed<br>17. know whom to turn to, if help is needed<br>18. can stay outside home with friends and acts independently of family
**DECISION MAKING**
19.The patient take parts in the treatment related decisions
