# Supplementary material for: A Personalized eHealth Transition Concept for Adolescents With Inflammatory Bowel Disease: Design of Intervention
Source: JMIR Pediatr Parent. 2019 Apr 24;2(1):e12258. doi: 10.2196/12258 (PMC6715343; doi:10.2196/12258)
Supplement: Multimedia Appendix 2 [file pediatrics_v2i1e12258_app2.pdf]

**Multimedia Appendix 2: Transfer readiness checklist for the gastroenterological provider in adult care.**
Assessment of the patient´s transfer readiness at the first appointment in adult care setting.

| ITEMS | ANSWERS |
|---|---|
| **COMMUNICATION** | |
| 1.a The patient arrives to the consultation: | Alone / With parents/ With another relative |
| 1.b If the patient don´t arrive alone, to what extent does he/she communicate independently with you? | Completely independently / Mostly independently / Minimally independently / Not at all independently |
| 1.c How well has the patient prepared for the consultation? | Very well / To some extent / Very Limited extent / Not at all |
| **DISEASE KNOWLEDGE** | |
| How do you assess the patient's knowledge of:<br>2. current medication (name, dose, major side effects)<br>3. own disease course/history<br>4. what to do in case of a disease flare<br>5. how he/she renews prescriptions<br>6. how he/she can get in contact with the hospital<br>7. the impact of smoking, drugs and alcohol on health<br>8. where he/she can seek further knowledge about IBD | Satisfactory / Some knowledge / Limited knowledge / No knowledge |
| 9.The patient knows whom he/she can turn to if help is needed: | Yes / To some extent / Very Limited extent / Not at all |
| **MEDICAL ADHERENCE** | |
| 10. How do you assess the patient's general medical adherence? | Mark at a visual analog scale (Adherent; Non-adherent) |
| **QUALITY OF DELIVERED MATERIAL** | |
| 11. The quality of the information received from the pediatric department transferring the patient was: | Satisfying /Mostly satisfying/ Unsatisfied / Extremely unsatisfactory |
| **GENERAL TRANSFER ASSESSMENT** | |
| 12. To what extent is this patient prepared for adult centered care? | Mark at a visual analog scale (As prepared as possible; Not prepared at all) |
